# Editorial: Ushering in a new year and reaching out to the next generation of family doctors

**DOI:** 10.4102/safp.v62i1.5096

**Published:** 2020-02-13

**Authors:** Klaus B. von Pressentin

**Affiliations:** 1 Division of Family Medicine and Primary Care, Stellenbosch University, Cape Town, South Africa

This edition is the first with our new publisher, AOSIS, an established open-access scholarly publisher founded by the third editor of the *South African Family Practice* (SAFP) journal, Prof. Pierre de Villiers. The move towards AOSIS introduced a change in the SAFP’s journal style, with the print version of the journal published as four parts per year (as opposed to the previous format of six editions per year). The journal will still be published online at www.safpj.co.za, and accepted research papers will be published once copy-editing has been completed. Each of the four parts will continue to have an appropriate mix of content designed to the needs of our readership: the continuous professional development (CPD) section (contributed by the academic programmes and Rural Doctors’ Association of South Africa), research papers and the ‘Mastering your fellowship’ section aimed at helping registrars and their supervisors prepare for the national exit exam. This edition also includes part 1 of a new four-part series, entitled ‘National Health Insurance Unpacked’, by South African Academy of Family Physicians (SAAFP) president Prof. Bob Mash (in consultation with Dr Nicholas Crisp, a consultant for the National Health Insurance (NHI) Fund Office at the Ministry of Health in Pretoria). This series intends to provide up-to-date information on NHI and what is really planned (with a focus on addressing the gaps identified in the 2019 SAAFP survey^1^). Furthermore, we would like to inform authors, reviewers and readers of a revised policy per submission type, as accepted by the new editorial board (see Table 1).

**TABLE 1:** Revised editorial policy per submission type.

Contribution type	Description	Word count allowed	Review process
**Original Research (including scientific reviews)**	Report of original scientific research conducted in family medicine and primary care, ethical approval essential.	Up to 7000 words	Editor/assistant editor reviews the manuscript and, if it meets the journal’s scope and guidelines, assigns two reviewers (for full peer-review process).
**Letters to the Editor**	Short comment in response to publications in previous editions or related matters.	Up to 800 words	Editor/assistant editor reviews the manuscript.
**Scientific Letters**	Short scientific report of original research, ethical approval essential. Case studies can be submitted as scientific letters.	Up to 1800 words	Editor/assistant editor reviews the manuscript and, if it meets the journal’s scope and guidelines, assigns two reviewers (for full peer-review process).
**CPD articles**	Articles are published as part of the continuous professional development (CPD) programme of SAFP.	Up to 1800 words	Editor/assistant editor invites authors to contribute. Articles are internally reviewed by the editors.
**Open Forum**	These contributions may discuss topical issues within the field of family medicine and primary care; innovations in clinical practice or conference reports.	Up to 1800 words	Editor/assistant editor reviews the manuscript and assigns additional reviewer(s) if required.
**Editorials**	Scientific editorials can be used to highlight progress in any scientific field related to family medicine and primary care.	Up to 800 words	Guest editorials on invitation basis; the SAAFP president is entitled to one editorial per year. The remaining editorials are divided between the editor and the assistant editor.

SAAFP, South African Academy of Family Physicians.

When I reached out to junior medical officers recently to encourage them to apply for a vacant family medicine registrar post, I was struck by their perceived lack of understanding of family medicine and primary care as a career option. The root causes may be numerous, but I was reminded of recent research^2,3,4,5^ which showed that the undergraduate exposure in medical curricula to our discipline seems to be insufficient. Ambivalence regarding the effect of students on service provision may hamper the process of extending primary care contact periods. A publication by members of the Collaboration for Health Equity through Education and Research (CHEER) concluded that undergraduate students can add benefit to health services if health professions educators plan their clinical rotations, with the proviso of recognising the pressures under which their clinical supervisors work to deliver services to patients.^6^ Significantly, the presiding policy perspective includes a call to transform the training of graduates and to align curricula with the international focus on social accountability and universal health coverage. This may be achieved through various methods, such as enrolling medical officers for a postgraduate primary care diploma^7^, training family physicians to scale^8^, as well as embracing the Academy of Science of South Africa (ASSAf) recommendations for socially accountable education.^9^ South Africa is not alone in this challenge of realising these policy directives, as voiced by Prof. Val Wass, editor of *Education for Primary Care* and an international member of the SAFP editorial board, in her recent editorial:

The global challenge of a clinical workforce unable to meet twenty-first century healthcare needs has arrived. I bemoan, once again, our tardy response to the 2010 Lancet report and the slow transition of learning into the primary care context.^10^

Curricular design and the presence of generalist primary care faculty in core teaching have crucial influences on career choices of medical students and young doctors.^11^ Revising medical curricula takes time; consequently, we should start by making the most of the short stints of undergraduate exposure to our primary care context. At the start of the New Year, I would like to encourage you to reach out to your team members, especially junior colleagues and students, and help structure the primary care space you share into an environment that encourages transformational learning experiences. Simple actions may be very effective, such as reflecting together on a difficult consultation from the patient’s perspective, arranging for the student to experience a mobile clinic or community health worker visit, or showing your human side by merely asking what inspires the student about medicine and listening with full attention. Such spontaneous and authentic moments in the everyday reality of our busy context have the potential of showcasing the values and principles of our discipline. And, often, your love for primary care may be rejuvenated by experiencing your familiar work environment through fresh perspectives. Students and junior colleagues have an enormous advocacy potential for our discipline, which we should tap into. Culture can shift: the student’s voice needs to be heard (and read in this journal). Medical students and registrars are encouraged to offer their perspectives on family medicine and on their experiences as consumers of primary care curricular offerings. I trust that these ideas may motivate the SAFP journal readership to help build our discipline by facilitating meaningful primary care learning experiences and conversations.

Best wishes,

Klaus B. von Pressentin

Editor-in-Chief
